# Puberty, but not precocious puberty is influenced by weight gain in the first years of life

**DOI:** 10.1007/s12020-025-04254-3

**Published:** 2025-05-21

**Authors:** Vittorio Ferrari, Alessandra Li Pomi, Daniele Ciofi, Malgorzata Gabriela Wasniewska, Stefano Stagi

**Affiliations:** 1 Pediatric Unit, IRCCS Azienda Ospedaliero-Universitaria di Bologna, Bologna, Italy; 2 Department of Medical and Surgical Sciences, University of Bologna, Bologna, Italy; 3 Department of Human Pathology of Adulthood and Childhood, University of Messina, Messina, Italy; 4 Meyer Children’s Hospital IRCCS, Florence, Italy; 5 Pediatric Unit, Gaetano Martino University Hospital, Messina, Italy; 6 Department of Health Sciences, University of Florence, Florence, Italy

**Keywords:** Puberty, Precocious puberty, Menarche, Thelarche, Postnatal weight gain, Secular trend

## Abstract

**Introduction:**

Factors favouring the secular trend of decreasing average age of puberty include eating habits, environmental endocrine disruptors, genetics, stress and lifestyle. The association between higher BMI and timing and tempo of puberty has long been documented in the general population but data for children with precocious puberty are poor.

**Aims:**

To evaluate the relationship between the role of weight gain in the first years of life on the onset of central precocious puberty.

**Patients and Methods:**

We analyzed the data of 120 Caucasian girls diagnosed with CPP between May 2020 and March 2021 (group 1) and a control group of 540 girls. Patients with CPP associated with hypothalamic–pituitary congenital malformations, neurological, neurosurgical and/or genetic diseases, psychomotor delay, oncological diseases, other endocrine impairments requiring hormonal treatments, or taking drugs that may interfere with pubertal development were excluded.

**Results:**

In CPP girls (group 1), mean age of B2 was 7.67 ± 0.88 years; BMI was 0.14 ± 0.88 SDS, and average BW was −0.08 ± 1.04 SDS. In this group an evaluation of *delta SDS BMIB2-BW* did not reveal a statistically significant relationship between thelarche and increased BMI. In comparison, the mean age of B2 in the control group (group 2) was 10.06 ± 1.03 years, BMI was −0.02 ± 1.01 SDS, and mean BW was −0.03 ± 0.93 SDS and we found a clear correlation between delta *SDS BMIB2-BW* and thelarche age (R: 0.27; *p* < 0.0001).

**Conclusions:**

Our data confirm that weight gain plays a crucial role in the trend of earlier pubertal development in the general population, but precocious puberty does not appear to be influenced by weight variation in the first years of life. It is therefore important to consider other factors which may contribute to triggering or aggravating this condition.

## Introduction

Puberty is the developmental stage during which secondary sexual characteristics and reproductive capacity are acquired. Over the last few decades, there has been a progressive decrease in the age of pubertal development, especially in girls [1–3]. The factors involved include eating habits, environmental endocrine disruptors, genetics, stress and other lifestyle factors [1, 2, 4, 5].

During the Sars-CoV2 pandemic in Italy, especially in the months of lockdown from March to May 2020, an increase in cases of central precocious puberty (CPP) and rapidly progressing puberty was observed [6–8]. The data suggest that environmental factors, such as nutrition and excessive use of electronic devices, play a role in the early onset of pubertal development [6, 9–12].

The association between higher BMI and earlier onset of puberty has long been known. Increased adiposity is associated with an earlier onset of breast buttons [13–16] and menarche [13, 17] in girls and with an acceleration of pubertal progression [6].

In investigating how BMI affects the process of pubertal development, researchers have focused on the endocrine activity of white adipose tissue, including the production of adipokines and the conversion of androgens to estrogens via the enzyme aromatase [18]. Some studies indicate that important increases in BMI during the first 20 months of life [16], in the first five years [19] and between two and eight years of age [20] are associated with an earlier onset of puberty. Furthermore, there seems to be a relationship between birth weight (BW) and age at puberty [19, 21]. Insulin resistance and excess hepato-visceral fat may play an important role in determining the appearance of the first signs of pubertal development. The early appearance of pubertal development could therefore be a mechanism to minimize increases in central ectopic fat [22]. Variations between BW and childhood BMI could represent a marker for predicting the timing and tempo of puberty [22].

A recent study on a cohort of 577 girls in central Italy showed that a greater variation between BW and Z-score of BMI during childhood corresponds to an earlier onset of puberty and a greater speed of development (defined as a shorter time between thelarche and menarche) [13].

There is no clear evidence regarding the impact of weight gain in the first years of life on the onset of central precocious puberty (CPP). Some studies indicate that a prolonged period of obesity in the first years of life is associated with CPP [23, 24].

Our study aims to evaluate whether these is a correlation between weight gain from birth to the appearance of secondary sexual characteristic and central precocious puberty.

## Patients and methods

We carried out a monocentric, retrospective and observational study. We analyzed the data of 120 Caucasian girls diagnosed with CPP by the Auxoendocrinology and Gynecology Unit of Meyer Children Hospital between May 2020 and March 2021 (group 1) and a control group of 540 girls (group 2) who had been referred to the same Operating Unit during the same period for suspected CPP but who did not meet the criteria to receive this diagnosis.

Patients with a diagnosis of CPP and associated hypothalamic–pituitary congenital malformations, neurological, neurosurgical and/or genetic diseases, psychomotor delay, oncological diseases, other endocrine impairments requiring hormonal treatments, or taking drugs that may interfere with pubertal development were excluded from the study. We also excluded children who were born small for gestational age (SGA: birth weight and/or birth length less than 2 SDS below the mean for gestational age) [25], were adopted or had immigrated to Italy as such children have a statistically higher rate of precocious puberty than the general pediatric population.

The study was performed according to the Helsinki II declaration and approved by the local Paediatric Ethical Committee (Paediatric Ethical Committee – Tuscany Region, approval number: 65/2019, in date 03/09/2019). Written informed consent was obtained from the parents of enrolled children.

### Study design

All patients underwent periodical auxological examinations during which weight, height, body mass index (BMI) and pubertal progression rate (Tanner scale) were recorded [26]. Birth weight (BW) was collected from the patient’s pediatric booklet. BMI was calculated by dividing the patient’s weight in kilograms by the square of height in meters [27]. Height and BMI were normalized for chronological age by conversion to SD scores [26].

Pubertal development was classified according to the Marshall and Tanner criteria [28]. The age of pubertal onset was defined as the age at durable Tanner B2 stage, confirmed by auxological, endocrinological and/or radiological results [29].

Precocious puberty was defined as the development of pubertal changes at an age that was younger than the accepted lower limits for onset of puberty (before the age of 8 years in girls) [30]. We considered peak LH values of > 5 IU/L on the GnRH stimulation test in the presence of pubertal signs or a basal LH value of > 1.1 IU/L and a ratio of stimulated LH to stimulated FSH of > 1.0 combined with isolated pubic and/or axillary hair growth accompanied by breast development [30] to be indicative of activation of the hypothalamic GnRH pulse generator.

### Auxological evaluation

Height was measured using Harpenden’s stadiometer in triplicate to the nearest 0.1 cm. Weight was determined to the nearest 0.1 kg using a balance scale. To calculate the standard deviation scores of the neonatal data (weight and length at birth) we considered the Italian Neonatal Study [INeS] charts [31]; for auxological parameters (height, weight and BMI at the time of thelarche) we used the Italian cross sectional growth charts of Cacciari et al. [32]. Girls with a BMI above the 85th centile (+1 SDS) were considered overweight [33]. To evaluate the influence of the accumulation of central ectopic fat on the age of pubertal development we used the formula described by de Zegher et al. [22, 34].

Within the group of girls diagnosed with CPP, we calculated the delta SDS between BMI at the onset of B2 and birth weight (*delta SDS BMIB2-BW*) [22] and subsequently looked for any correlation between this value and the age of onset of pubertal development (attainment of Tanner stage B2).

### Statistical analysis

Statistical analyses were performed with SPSS X software (SPSSX Inc., Chicago, IL, USA). The characteristics of the study population were described using frequency distributions for categorical variables and mean and standard deviation (SD) values, medians, and ranges for continuous variables, depending on whether the data were normally distributed. The statistical significance of the continuous variable comparisons was assessed using the Student t test and the Mann-Whitney U test, depending on the distribution of the analyzed variable; the comparison of categorical variables was conducted using the chi square test or Fisher’s Exact test if there was a small (<5) expected cell size. The Pearson correlation test was used to determine the correlation coefficients. All statistical tests were two-tailed and a *p* < 0.05 was considered statistically significant.

## Results

The mean age at the onset of B2 within girls diagnosed with CPP (group 1) was 7.67 ± 0.88 years; the mean BMI of this group was 17.43 ± 2.29 kg/m^2^ (0.14 ± 0.88 SDS), average BW was 3097.150 ± 507.179 gr (−0.08 ± 1.04 SDS) and the mean height was 129.99 ± 7.58 cm (0.60 ± 0.94 SDS). Evaluating the *delta SDS BMIB2-BW*, we did not observe any statistically significant relationship between these values (R: 0.037; *p* = 0.85) (Table 1).

**Table 1:** Main results observed in the two study groups

	Subjects (n°)	Mean age at B2 (years)	BW (g)	SDS BW^a^	BMI at B2 (kg/m^2^)	SDS BMI at B2^b^	Correlation coefficient (R)	*P* value
**Group 1**	120	7.67 ± 0.88	3097.150 ± 507.179	−0.08 ± 1.04	17.43 ± 2.29	0.14 ± 0.88	0.037	0.85
**Group 2**	540	10.06 ± 1.03	3182.600 ± 460.458	−0.03 ± 0.93	18.57 ± 2.94	−0.02 ± 1.01	0.27	<0.0001

^a^According to the the Italian Neonatal Study [INeS] charts [28]

^b^According to the Italian cross-sectional growth charts of Cacciari et al. [29]

**Fig. 1:**
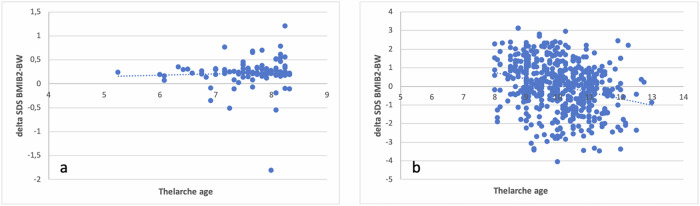
Correlation between delta SDS BMIB2-BW and thelarche age in: **a** girls diagnosed with CPP (group 1; R: 0.037; *p* = 0.85); **b** control group (group 2; R: 0.27; *p* < 0.0001

The mean age of onset of B2 for girls in the control group (group 2) was 10.06 ± 1.03 years, whereas mean BMI was 18.57 ± 2.94 kg/m^2^ (−0.02 ± 1.01 SDS), mean BW was 3182.600 ± 460.458 gr (−0.03 ± 0.93 SDS) and average height was 141.00 ± 7.57 cm (0.29 ± 1.03 SDS) Fig. 1. In this group, we observed a statistically significant relationship between delta *SDS BMIB2-BW* and age at thelarche (R: 0.27; *p* < 0.0001), confirming findings previously reported by *Ferrari* et al. [13].

Among the overweight girls in group 1 (18 out of 120 patients, 15%), mean age at onset of pubertal development was 7.29 ± 1.7 years, with a mean height of 129.64 ± 12.92 cm (0.89 ± 1.03 SDS), an average BW of 3763.666 ± 354.795 gr (0.37 ± 1.44 SDS) and an average BMI of 21.38 ± 2.03 kg/m^2^ (1.41 ± 0.33 SDS). In this subgroup we did not observe a statistically significant intercurrent relationship between *delta SDS BMIB2-BW* and age at thelarche (R: 0.094; *p* = 0.81), although the small number of girls in this group must be taken into account.

## Discussion

Our data confirm the influence of weight change from birth to the time of Tanner stage B2 (*delta SDS BMIB2-BW*) on the age of pubertal development in healthy girls as previously observed in a study by Ferrari et al. [13].

We did not observe the same relationship in the group of girls diagnosed with CPP, and we thus hypothesize that other factors play a crucial role in the development of this condition.

Even in the overweight girls of the group, there would was no association between pubertal development and weight variation from birth. However, we acknowledge that this subgroup was small. In fact, in our cohort of girls diagnosed with CPP only 18 out of 120 (15%) were overweight at the time of diagnosis.

Our study suggests that precocious puberty is not triggered or aggravated by weight variation in the first years of life.

These observations are in contrast with findings in healthy children in the general population in whom weight gain does lead to earlier onsets of puberty [13–16]. Moreover, SGA patients also show an age at menarche earlier and a pubertal progression slightly faster than general population, related also to a premature adrenarche and an accelerated weight gain during early childhood [25].

The interaction between adipokines and neuronal circuits involved in regulating the timing of pubertal development has been extensively studied. As far as we know, the adipokines produced by adipose tissue are among the main regulators of eating behavior and carry out their role by acting on the hypothalamus. The leptin-kisspeptin axis is the most fully understood of the interaction systems between adipokines and neuronal networks [18]. Leptin is an appetite suppressant hormone that regulates the level of body mass in the long term and operates by turning off hunger neurons at the hypothalamic level [35]. After binding to its receptor, it causes the activation of kisspeptin-secreting neurons, that inform the body that there are sufficient energy reserves to start a period of fertility. It has been shown that leptin concentration increases before the onset of puberty in girls and a leptin peak precedes that of gonadotropins [36, 37]. Also noteworthy is the negative correlation found by Matkovic et al. between age of menarche and leptin concentration in girls [38].

Another metabolic regulator which behaves in the opposite way to leptin is ghrelin, which is secreted when the stomach is empty and when there is a negative metabolic balance [39]. Ghrelin depresses kisspeptin expression at the arcuate nucleus of the hypothalamus [39].

We also know that receptors for adiponectin are present at hypothalamic, pituitary and gonadal levels. Adiponectin inhibits the activity of GnRH neurons via AMP kinase pathways and has an insulin-sensitizing action [40]. Its lower concentration in obese patients compared to healthy controls could play a role in the earlier onset of puberty in the former [41].

We have been aware of the association between increased BMI and hyperinsulinemia for a long time [42] and we know that elevated levels of insulinemia are associated with a reduction in the concentration of sex-hormone-binding-globulins. This would determine a greater bioavailability of sex hormones [43] and consequently it could influence the timing of pubertal development.

Our study suggests that weight gain plays a crucial role in the trend of earlier puberty in the general population, but that there are other important factors at play in patients with real precocious puberty. These might include genetics, endocrine disruptors and lifestyle habits (e.g. increased use of electronic devices) [6, 9–12].

In conclusion, it is undoubtedly necessary to conduct studies on larger cohorts of girls diagnosed with CPP to better understand the role of weight gain in the first years of life in the early development of sexual characteristics. At the same time, our study suggests that it is equally important to investigate other factors which may trigger or aggravate this pathological condition.

## Data Availability

No datasets were generated or analysed during the current study.

## References

[1] G. Farello, C. Altieri, M. Cutini, G. Pozzobon, A. Verrotti, Review of the literature on current changes in the timing of pubertal development and the incomplete forms of early puberty. Front. Pediatr. **7**, 147–153 (2019). 10.3389/fped.2019.00147. 31139600 10.3389/fped.2019.00147 PMC6519308

[2] V. Ferrari, S. Stefanucci, D. Ciofi, S. Stagi, Analysis of the timing of puberty in a recent cohort of Italian girls: evidence for earlier onset compared to previous studies. J. Pediatr. Adolesc. Gynecol. **35**(1), 23–29 (2021). 10.1016/j.jpag.2021.06.007. 34166823 10.1016/j.jpag.2021.06.007

[3] F.M. Biro, L.C. Greenspan, M.P. Galvez, Puberty in girls of the 21st century. J. Pediatr. Adolesc. Gynecol. **25**(5), 289–294 (2012). 10.1016/j.jpag.2012.05.009. 22841372 10.1016/j.jpag.2012.05.009 PMC3613238

[4] A.S. Parent, G. Teilmann, A. Juul, N.E. Skakkebaek, J. Toppari, J.P. Bourguignon, The timing of normal puberty and the age limits of sexual precocity: variations around the world, secular trends, and changes after migration. Endocr. Rev. **24**(5), 668–693 (2003). 10.1210/er.2002-0019. 14570750 10.1210/er.2002-0019

[5] K. Sorensen, A. Mouristen, L. Aksglaede, C.P. Hagen, S.S. Mogensen, A. Juul, Recent secular trends in pubertal timing: implications for evaluation and diagnosis of precocious puberty. Horm. Res. Paediatr. **77**(3), 137–145 (2012). 10.1159/000336325. 22508036 10.1159/000336325

[6] S. Stagi, S. De Masi, E. Bencini, S. Losi, S. Paci, M. Parpagnoli, F. Ricci, D. Ciofi, C. Azzari, Increased incidence of precocious and accelerated puberty in females during and after the Italian lockdown for the coronavirus 2019 (COVID-19) pandemic. Ital. J. Pediatr. **46**(1), 165–174 (2020). 10.1186/s13052-020-00931-3. 33148304 10.1186/s13052-020-00931-3 PMC7609833

[7] M. Peinkhofer, B. Bossini, A. Penco, M. Giangreco, M.C. Pellegrin, V. Vidonis, G. Vittori, N. Grassi, E. Faleschini, E. Barbi, G. Tornese, Reduction in pediatric growth hormone deficiency and increase in central precocious puberty diagnoses during COVID 19 pandemics. Ital. J. Pediatr. **48**(1), 49 (2022). 10.1186/s13052-022-01238-1. 35346309 10.1186/s13052-022-01238-1 PMC8960104

[8] Turriziani Colonna, A.; Curatola, A.; Sodero, G.; Lazzareschi, I.; Cammisa, I.; Cipolla, C. Central precocious puberty in children after COVID-19 outbreak: a single-center retrospective study. Minerva Pediatr. (2022) 10.23736/S2724-5276.22.06827-6 10.23736/S2724-5276.22.06827-6 35586885

[9] M. Verzani, C. Bizzarri, L. Chioma, G. Bottaro, S. Pedicelli, M. Cappa, Impact of COVID-19 pandemic lockdown on early onset of puberty: experience of an Italian tertiary center. Ital. J. Pediatr. **47**(1), 52–54 (2021). 10.1186/s13052-021-01015-6. 33673836 10.1186/s13052-021-01015-6 PMC7935003

[10] L. Chioma, C. Bizzarri, M. Verzani, D. Fava, M. Salerno, D. Capalbo, C. Guzzetti, L. Penta, L. Di Luigi, N. di Iorgi, M. Maghnie, S. Loche, M. Cappa, Sedentary lifestyle and precocious puberty in girls during the COVID-19 pandemic: an Italian experience. Endocr. Connect. **11**(2), e210650 (2022). 10.1530/EC-21-0650. 35029543 10.1530/EC-21-0650 PMC8859940

[11] M. Goffredo, A. Pilotta, I. Parissenti, C. Forino, C. Tomasi, P. Goffredo, F. Buzi, R. Badolato, Early onset of puberty during COVID-19 pandemic lockdown: experience from two pediatric endocrinology Italian centers. J. Pediatr. Endocrinol. Metab. **36**, 290–298 (2023). 10.1515/jpem-2022-0492. 36607833 10.1515/jpem-2022-0492

[12] M.E. Street, D. Ponzi, R. Renati, M. Petraroli, T. D’Alvano, C. Lattanzi, V. Ferrari, D. Rollo, S. Stagi, Precocious puberty under stressful conditions: new understanding and insights from the lessons learnt from international adoptions and the COVID-19 pandemic. Front. Endocrinol. **14**, 1149417 (2023). 10.3389/fendo.2023.1149417. 10.3389/fendo.2023.1149417 PMC10187034 37201098

[13] V. Ferrari, S. Stefanucci, M. Ferrari, D. Ciofi, S. Stagi, on the behalf of the Tuscany Menarche Study Group, Retrospective longitudinal analysis of the effects of postnatal weight gain on the timing and tempo of puberty and menarche in a cohort of Italian girls. Ital. J. Pediatr. **48**(1), 20 (2022). 10.1186/s13052-022-01222-9. 35115014 10.1186/s13052-022-01222-9 PMC8811590

[14] P.B. Kaplowitz, E.J. Slora, R.C. Wasserman, S.E. Pedlow, M.E. Herman-Giddens, Earlier onset of puberty in girls: relation to increased body mass index and race. Pediatrics **108**, 347–353 (2001). 10.1542/peds.108.2.347. 11483799 10.1542/peds.108.2.347

[15] H.M. Ma, M.L. Du, X.P. Luo, S.K. Chen, L. Liu, R.M. Chen, C. Zhu, F. Xiong, T. Li, W. Wang, G.L. Liu, Pubertal Study Group of the Society of Pediatric Endocrinology and Genetic Disease, Chinese Medical Association, Onset of breast and pubic hair development and menses in urban chinese girls. Pediatrics **124**(2), e269–e277 (2009). 10.1542/peds.2008-2638. 19651567 10.1542/peds.2008-2638

[16] M. Maisonet, K.Y. Christensen, C. Rubin, A. Holmes, W.D. Flanders, J. Heron, K.K. Ong, J. Golding, M.A. McGeehin, M. Marcus, Role of prenatal characteristics and early growth on pubertal attainment of British girls. Pediatrics **126**(3), e591–e600 (2010). 10.1542/peds.2009-2636. 20696722 10.1542/peds.2009-2636 PMC5578444

[17] D.S. Freedman, L.K. Khan, M.K. Serdula, W.H. Dietz, S.R. Srinivasan, G.S. Berenson, Relation of age at menarche to race, time period, and anthropometric dimensions: the bogalusa heart study. Pediatrics **110**, e43 (2002). 10.1542/peds.110.4.e43. 12359816 10.1542/peds.110.4.e43

[18] C. Bruno, E. Vergani, M. Giusti, A. Oliva, C. Cipolla, D. Pitocco, A. Mancini, The “Adipo-Cerebral” dialogue in childhood obesity: focus on growth and puberty. physiopathological and nutritional aspects. Nutrients **13**(10), 3434 (2021). 10.3390/nu13103434. 34684432 10.3390/nu13103434 PMC8539184

[19] Y. Wang, G.E. Dinse, W.J. Rogan, Birth weight early weight gain and pubertal maturation: a longitudinal study. Pediatr. Obes. **7**(2), 101–109 (2012). 10.1111/j.2047-6310.2011.00022.x. 22434749 10.1111/j.2047-6310.2011.00022.x PMC3313082

[20] Q. He, J. Karlberg, Bmi in childhood and its association with height gain, timing of puberty, and final height. Pediatr. Res. **49**, 244–251 (2001). 10.1203/00006450-200102000-00019. 11158521 10.1203/00006450-200102000-00019

[21] N. Karaolis-Danckert, A.E. Buyken, A. Sonntag, A. Kroke, Birth and early life infuences on the timing of puberty onset: results from the DONALD (DOrtmund Nutritional and Anthropometric Longitudinally Designed) study. Am. J. Clin. Nutr. **90**, 1559–1565 (2009). 10.3945/ajcn.2009.28259. 19828713 10.3945/ajcn.2009.28259

[22] F. de Zegher, L. Ibanez, On the rising incidence of early breast development: puberty as an adaptive escape from ectopic adiposity in mismatch girls. Eur. J. Endocrinol. **185**(1), L1–L2 (2021). 10.1530/EJE-21-0287. 33950862 10.1530/EJE-21-0287 PMC8183632

[23] G. Liu, J. Guo, X. Zhang, Y. Lu, J. Miao, H. Xue, Obesity is a risk factor for central precocious puberty: a case-control study. BMC Pediatr. **21**(1), 509 (2021). 10.1186/s12887-021-02936-1. 34784914 10.1186/s12887-021-02936-1 PMC8594221

[24] Y. Choe, J.H. Cha, Y.J. Kim, J. Choi, K. Lee, N. Kim, J.Y. Na, S. Yang, Rapid weight gain in early life is associated with central precocious puberty in girls, not in boys - a nationwide population-based study in Korea. Front. Endocrinol. **14**, 1210995 (2023). 10.3389/fendo.2023.1210995. 10.3389/fendo.2023.1210995 PMC10381025 37522114

[25] A.C.S. Hokken-Koelega, M. van der Steen, M.C.S. Boguszewski, S. Cianfarani, J. Dahlgren, R. Horikawa, V. Mericq, R. Rapaport, A. Alherbish, D. Braslavsky, E. Charmandari, S.D. Chernausek, W.S. Cutfield, A. Dauber, A. Deeb, W.J. Goedegebuure, P.L. Hofman, E. Isganatis, A.A. Jorge, C. Kanaka-Gantenbein, K. Kashimada, V. Khadilkar, X.P. Luo, S. Mathai, Y. Nakano, M. Yau, International consensus guideline on small for gestational age: etiology and management from infancy to early adulthood. Endocr. Rev. **44**(3), 539–565 (2023). 10.1210/endrev/bnad002. 36635911 10.1210/endrev/bnad002 PMC10166266

[26] S. Stagi, L. Galli, C. Cecchi, E. Chiappini, S. Losi, C.G. Gattinara, C. Gabiano, P.A. Tovo, S. Bernardi, F. Chiarelli, M. de Martino, Final height in patients perinatally infected with the human immunodeficiency virus. Horm. Res. Paediatr. **74**(3), 165–171 (2010). 10.1159/000281018. 20516649 10.1159/000281018

[27] S. Stagi, F. Ricci, M. Bianconi, M.A. Sammarco, G. Municchi, S. Toni, L. Lenzi, A. Verrotti, M. de Martino, Retrospective evaluation of metformin and/or metformin plus a new polysaccharide complex in treating severe Hyperinsulinism and insulin resistance in obese children and adolescents with metabolic syndrome. Nutrients **9**(5), 524 (2017). 10.3390/nu9050524. 28531113 10.3390/nu9050524 PMC5452254

[28] W.A. Marshall, J.M. Tanner, Variations in patterns of pubertal changes in girls. Arch. Dis. Child. **44**(235), 291–303 (1969). 10.1136/adc.44.235.291. 5785179 10.1136/adc.44.235.291 PMC2020314

[29] T. Karatzias, M. Shevlin, J. Murphy, O. McBride, M. Ben-Ezra, R.P. Bentall, F. Vallières, P. Hyland, Posttraumatic stress symptoms and associated comorbidity during the COVID-19 pandemic in Ireland: a population-based study. J. Trauma. Stress. **33**(4), 365–370 (2020). 10.1002/jts.22565. 32662129 10.1002/jts.22565 PMC7405473

[30] J.C. Carel, E.A. Eugster, A. Rogol, L. Ghizzoni, M.R. Palmert, ESPE-LWPES GnRH analogs consensus conference group, F. Antoniazzi et al., Consensus statement on the use of gonadotropin-releasing hormone analogs in children. Pediatrics **123**(4), e752–e762 (2009). 10.1542/peds.2008-1783. 19332438 10.1542/peds.2008-1783

[31] E. Bertino, P. Di Nicola, A. Varalda, L. Occhi, F. Giuliani, A. Coscia, Neonatal growth charts. J. Matern. Fetal Neonatal Med. **25**(Suppl 1), 67–69 (2012). 10.3109/14767058.2012.664889. 22348405 10.3109/14767058.2012.664889

[32] E. Cacciari, S. Milani, A. Balsamo, E. Spada, G. Bona, L. Cavallo, F. Cerutti, L. Gargantini, N. Greggio, G. Tonini, A. Cicognani, Italian cross-sectional growth charts for height, weight and BMI (2 to 20 yr). J. Endocrinol. Invest. **29**(7), 581–593 (2006). 10.1007/BF03344156. 16957405 10.1007/BF03344156

[33] C.B. Weir, A. Jan, BMI classification percentile and cut off points. in: StatPearls. (Treasure Island (FL), 2023). StatPearls Publishing; 2025 31082114

[34] F. de Zegher, R. Malpique, C. Garcia-Beltran, L. Ibanez, Towards a simple marker of hepato-visceral adiposity and insulin resistance: the Z-score change from weight-at-birth to BMI-in-childhood. Pediatr. Obes. **14**(10), e12533 (2019). 10.1111/ijpo.12533. 31184433 10.1111/ijpo.12533

[35] D.A. de Luis, J.L. Perez Castrillón, A. Dueñas, Leptin and obesity. Minerva Med. **100**, 229–236 (2009). 19182739

[36] J.N. Roemmich, P.A. Clark, S.S. Berr, V. Mai, C.S. Mantzoros, J.S. Flier, A. Weltman, A. Rogol, Gender differences in leptin levels during puberty are related to the subcutaneous fat depot and sex steroids. Am. J. Physiol. **275**, E543–E551 (1998). 9725824 10.1152/ajpendo.1998.275.3.E543

[37] F. Rutters, A.G. Nieuwenhuizen, S.P.M. Verhoef, S.G.T. Lemmens, N. Vogels, M.S. Westerterp-Plantenga, The relationship between leptin, gonadotropic hormones, and body composition during puberty in a Dutch children cohort. Eur. J. Endocrinol. **160**, 973–978 (2009). 19332528 10.1530/EJE-08-0762

[38] V. Matkovic, J.Z. Ilich, M. Skugor, N.E. Badenhop, P. Goel, A. Clairmont, D. Klisovic, R.W. Nahhas, J.D. Landoll, Leptin is inversely related to age at menarche in human females. J. Clin. Endocrinol. Metab. **82**, 3239–3245 (1997). 9329346 10.1210/jcem.82.10.4280

[39] K.C. Anderson, F. Hasan, E.E. Grammer, S. Kranz, Endogenous ghrelin levels and perception of hunger: a systematic review and meta-analysis. Adv. Nutr. **14**(5), 1226–1236 (2023). 10.1016/j.advnut.2023.07.011. 37536563 10.1016/j.advnut.2023.07.011 PMC10509419

[40] H. Mathew, V.D. Castracane, C. Mantzoros, Adipose tissue and reproductive health. Metabolism **86**, 18–32 (2018). 29155136 10.1016/j.metabol.2017.11.006

[41] Y. Arita, S. Kihara, N. Ouchi, M. Takahashi, K. Maeda, J.I. Miyagawa, K. Hotta, I. Shimomura, T. Nakamura, K. Miyaoka et al., Paradoxical decrease of an adipose-specific protein, adiponectin, in obesity. Biochem. Biophys. Res. Commun. **257**, 79–83 (1999). 10092513 10.1006/bbrc.1999.0255

[42] N.M. Templeman, S. Skovso, M.M. Page, G.E. Lim, J.D. Johnson, A causal role for hyperinsulinemia in obesity. J. Endocrinol. **232**, R173–R183 (2017). 28052999 10.1530/JOE-16-0449

[43] K. Sørensen, L. Aksglaede, T. Munch-Andersen, N.J. Aachmann-Andersen, J.H. Petersen, L. Hilsted, J.W. Helge, A. Juul, Sex hormone-binding globulin levels predict insulin sensitivity, disposition index, and cardiovascular risk during puberty. Diabetes Care **32**, 909–914 (2009). 19196890 10.2337/dc08-1618 PMC2671098

